# Function of B-Cell CLL/Lymphoma 11B in Glial Progenitor Proliferation and Oligodendrocyte Maturation

**DOI:** 10.3389/fnmol.2018.00004

**Published:** 2018-01-24

**Authors:** Chih-Yen Wang, Yuan-Ting Sun, Kuan-Min Fang, Chia-Hsin Ho, Chung-Shi Yang, Shun-Fen Tzeng

**Affiliations:** ^1^Institute of Life Sciences, College of Bioscience and Biotechnology, National Cheng Kung University, Tainan, Taiwan; ^2^Department of Neurology, National Cheng Kung University Hospital, College of Medicine, National Cheng Kung University, Tainan, Taiwan; ^3^Institute of Biomedical Engineering and Nanomedicine, National Health Research Institutes, Zhunan, Taiwan

**Keywords:** Bcl11b, glia, oligodendrocytes, glial progenitors, differentiation and proliferation

## Abstract

B-cell CLL/lymphoma 11B (Bcl11b) – a C2H2 zinc finger transcriptional factor – is known to regulate neuronal differentiation and function in the development of the central nervous system (CNS). Although its expression is reduced during oligodendrocyte (OLG) differentiation, its biological role in OLGs remains unknown. In this study, we found that the downregulation of Bcl11b gene expression in glial progenitor cells (GPCs) by lentivirus-mediated gene knockdown (KD) causes a reduction in cell proliferation with inhibited expression of stemness-related genes, while increasing the expression of cell cyclin regulator p21. In contrast, OLG specific transcription factors (Olig1) and OLG cell markers, including myelin proteolipid protein (PLP) and myelin oligodendrocyte glycoprotein (MOG), were upregulated in Bcl11b-KD GPCs. Chromatin immunoprecipitation (ChIP) analysis indicated that Bcl11b bound to the promoters of Olig1 and PLP, suggesting that Bcl11b could act as a repressor for Olig1 and PLP, similar to its action on p21. An increase in the number of GC^+^- or PLP^+^- OLGs derived from Bcl11b-KD GPCs or OLG precursor cells was also observed. Moreover, myelin basic protein (MBP) expression in OLGs derived from Bcl11b-KD GPCs was enhanced in hippocampal neuron co-cultures and in cerebellar brain-slice cultures. The *in vivo* study using a lysolecithin-induced demyelinating animal model also indicated that larger amounts of MBP^+^-OLGs and PLP^+^-OLGs derived from implanted Bcl11b-KD GPCs were present at the lesioned site of the white matter than in the scramble group. Taken together, our results provide insight into the functional role of Bcl11b in the negative regulation of GPC differentiation through the repression of OLG differentiation-associated genes.

## Main Points:

(1) Bcl11b regulates glial progenitor proliferation via inhibition of cell cycle regulator p21.
(2) Bcl11b downregulation in glial progenitors promotes their differentiation into mature oligodendrocytes *in vitro* and *in vivo*.
(3) Bcl11b could bind to the promotor regions of cell cycle regulator p21, Olig1, and PLP1 to control the proliferation and differentiation of glial progenitors.

## Introduction

Oligodendrocytes (OLGs), myelin-producing glia cells in the CNS, not only support the structure and energy metabolism of axons, but also facilitate the propagation of action potentials by extending their cellular processes to form multilayered myelin sheaths (Nave and Werner, 2014; Kremer et al., 2016). OLGs have been known to arise from OLG precursor cells (OPCs) during development and in adults (Herrera et al., 2001; Zuchero and Barres, 2013; Mayoral and Chan, 2016; Yang et al., 2017). In general, in the early phase of OLG differentiation, pre-myelinating OLGs with numerous complex elongating processes are generated from OPCs, and express O4, 2′,3′-cyclic-nucleotide 3′-phosphodiesterase (CNPase) and galactocerebroside (GC) (Zhang, 2001). The pre-myelinating OLGs further differentiate into mature myelinating OLGs, which are able to encircle axons with their extensions to form compact multilayered myelin consisting of lipids and myelin-associated proteins (Czepiel et al., 2015). The major myelin proteins in the CNS are MBP and PLP. Meanwhile, MOG and myelin-associated glycoprotein (MAG) only constitute 1% of all CNS myelin proteins (Nave and Werner, 2014). The differentiation of OPCs toward mature OLGs is controlled by extrinsic and transcriptional programs. It is well-documented that a pair of basic helix-loop-helix (bHLH) transcriptional factors, together with Olig1, Olig2, and Sox10, are required for OLG specification and maturation (Lu et al., 2002; Stolt et al., 2002; Zuchero and Barres, 2013). In addition, axonal/glial secreted factors and neuronal activity are important for OLG maturation and myelination (Emery, 2010; Zuchero and Barres, 2013). The progenitors isolated from embryonic and postnatal CNS tissues have been reported to give rise to astrocytes in serum-containing medium, but differentiate into OLGs in defined medium (Raff et al., 1983; Yang et al., 2011, 2017). For this reason, such bipotential progenitor cells have been termed as glial progenitor cells (GPCs). Accordingly, GPCs and OPCs are widely used as the culture models to study the molecular regulation of oligodendrocyte differentiation.

B-cell chronic lymphocytic leukemia/lymphoma 11B (Bcl11b), also named Ctip2, is a C2H2 zinc finger protein, originally discovered in T lymphoblastic leukemia (Bernard et al., 2001), and identified as a tumor suppressor in hematopoietic malignancies. The molecule can regulate cell-cycle progression by acting as a repressor for the expression of cyclin dependent kinase (CDK) inhibitors, such as p21 (Cherrier et al., 2009; Grabarczyk et al., 2010; Karanam et al., 2010; Chen et al., 2012). Expressed Bcl11b has been found in mouse brain regions, including the neocortex, hippocampus, and striatum (Arlotta et al., 2005, 2008; Chen et al., 2008; Simon et al., 2012), and is involved in the development of mouse cortical-projection neurons and striatal neurons (Arlotta et al., 2005, 2008; Chen et al., 2008). Findings indicating that proliferating progenitors and post-mitotic dentate granule cells in the dentate gyrus declined in Bcl11b mouse mutants during postnatal development point to the important role of Bcl11b in the regulation of progenitor proliferation (Simon et al., 2012). Moreover, the lack of Bcl11b-impaired post-mitotic neuron differentiation in the hippocampus of a developing mouse (Simon et al., 2012) demonstrates that progenitor cell proliferation and differentiation in the developing CNS depend on Bcl11b expression. Despite Bcl11b expression being significantly higher in neurons than that detected in OPCs (Zhang et al., 2014), its levels in newly formed OLGs and myelinating OLGs were much lower than in OPCs. However, the involvement of Bcl11b in glial differentiation has not yet been identified.

In this study, we provide evidence in a rat model for the Bcl11b-mediated regulation of OLG maturation by using commercially available rat GPCs and OPCs prepared from rat embryonic cortex. To determine the functional role of Bcl11b in GPCs/OPCs and OLGs, lentivirus-mediated knockdown of Bcl11b (Bcl11b-KD) was first performed to effectively reduce Bcl11b expression in GPCs. The downregulation of Bcl11b not only suppressed GPC cell proliferation, but also reduced their stemness markers. Interestingly, Bcl11b-KD increased the expression of OLG cell markers (PLP and MOG), as well as the number of OLGs in the cultures. Moreover, MBP expression in OLGs derived from Bcl11b-KD GPCs was increased in the presence of neurons, which possibly could enhance neuronal interaction with OLGs from the Bcl11b-KD GPCs. These observations were also verified by implanting Bcl11b-KD GPCs into the lysolecithin-treated corpus callosum of adult rat brains. We also infer that the manipulation of Bcl11b expression in GPCs/OPCs could foster their differentiation toward mature OLGs in demyelinating CNS tissues.

## Materials and Methods

### Materials

Media (DMEM/F12, MEM, Neurobasal medium), GlutaMAX^TM^, StemPro^®^ NSC SFM, B27 supplement, N2 supplement, poly-L-ornithine (PLO), and Lipofectamine 2000 were purchased from Invitrogen. Horse serum (HS) was obtained from HyClone Laboratories. Apotransferrin, biotin, bovine serum albumin (BSA), 5-bromo-2′-deoxyuridine (BrdU), cytosine arabinoside (Ara-C), diethylpyrocarbonate (DEPC), hydrocortisone, insulin, *N*-acetyl-cysteine (NAC), poly-D-lysine (PDL), sodium ampicillin, sodium pyruvate, sodium selenite, triiodothyronine (T3), and lysolecithin were obtained from Sigma. Ciliary neurotrophic factor (CNTF), epidermal growth factor (EGF), fibroblast growth factor-2 (FGF-2), and platelet derived growth factor-AA (PDGF-AA) were obtained from ProSpec. The antibodies used in this study are listed in **Table 1**.

**Table 1:** The antibodies used in the study.

Antibodies	Manufacturer	Immunogen	Working dilution
Monoclonal mouse anti-APC (clone CC1)	Calbiochem (OP80)	Recombinant protein consisting of amino acids 1–226 of APC	1:50 (IF)
Monoclonal rat anti-Bcl11b	Abcam (ab18465)	Fusion protein of human Ctip2 amino acids 1–150 [25B6]	1:500 (WB)
Polyclonal rabbit anti-Bcl11b	Abcam (ab28448)	Synthetic peptide within residues 850–950 of human Bcl11b	1:200 (IF for rat brain)
Polyclonal rabbit anti-Bcl11b	Novus (NBP2-33549)	Recombinant protein: QGNPQHLSQRELITPEADHVEAAILEEDEGLEIEEPSGLGLMVGGPDPDLLTCG	1:200 (IF for mouse brain)
Monoclonal mouse anti-CNP	Covance (SMI-91R)	46 and 48 kDa subunit of 94 kDa myelin CNPase dimer (SMI-91)	1:2000 (WB) 1:200 (IF)
Monoclonal mouse anti-GAPDH	Millipore (MAB374)	Glyceraldehyde-3-phosphate dehydrogenase from rabbit muscle	1:2000 (WB)
Polyclonal rabbit anti-GC	Millipore (MAB342)	Synaptic plasma membranes from bovine hippocampus	1:200 (WB)
Monoclonal mouse anti-GFP	Millipore (MAB2510)	Bacterially expressed GFP fusion protein	1:200 (IF)
Polyclonal rabbit anti-GFP	Millipore (MAB3080)	Highly purified native GFP from Aequorea victoria	1:200 (IF)
Monoclonal mouse anti-MBP	Calbiochem (NE1018)	Purified human myelin basic protein with amino acids 70–89	1:1000 (WB) 1:200 (IF)
Polyclonal rabbit anti-MOG	Abcam (ab32760)	Synthetic peptide as within residues 200 to C-terminal of rat myelin oligodendrocyte glycoprotein	1:1000 (WB) 1:200 (IF)
Polyclonal rabbit anti-NG2	Millipore (AB5320)	Immunoaffinity purified NG2 Chondroitin Sulfate Proteoglyacan from rat	1:200 (IF)
Polyclonal goat anti-NF200	Millipore (AB5539)	Purified bovine neurofilament-heavy	1:500 (IF)
Polyclonal rabbit anti-OLIG2	Millipore (AB9610)	Recombinant mouse Olig2	1:200 (IF)
Monoclonal mouse anti-p21	Calbiochem (OP79)	Recombinant mouse p21 protein (clone 22)	1:1000 (WB)
Rabbit antiserum anti-PDGFaR	Upstate (06-216)	GST-fusion protein corresponding to the 110 C-terminal amino acid residues of mouse PDGF type A receptor	1:200 (IF)
Polyclonal rabbit anti-PLP	Abcam (ab28486)	Synthetic peptide as amino acids 109–128 of mouse myelin PLP	1:1000 (WB) 1:200 (IF)

*IF, immunofluorescence; WB, western blot*.

#### Cell Culture

##### Rat glial progenitor cells (GPCs)

Glial progenitor cells prepared from cortical tissues of newborn Sprague-Dawley (SD) rats were purchased from Invitrogen (Cat no. N7746100). The cells, after passages, were seeded onto PLO-coated 100 mm petri dishes and maintained in the growth medium (GM) provided by the vendor (Wang et al., 2017). To induce the differentiation of GPCs into OLGs, the cultures were maintained for 5 days in OLG differentiation medium (DM), which contains basal components in the GM without growth factors.

##### Rat oligodendrocyte progenitor cells (OPCs)

Oligodendrocyte progenitor cells were prepared by modifying the protocol in (Fancy et al., 2012). Animal use followed the National Institutes of Health (NIH) Guidelines for Animal Research (Guide for the Care and Use of Laboratory Animals) and was approved by the National Cheng Kung University Institutional Animal Care and Use Committee, Tainan, Taiwan (IACUC approval number: 103060). Briefly, cortical tissues from SD rat embryos at 14.5 days were dissected and passed through a 40-μm pore filter. The cells were replated onto petri dishes without PDL coating, and cultured for 5–7 days in oligosphere medium – consisting of DMEM/F12 medium, 2% B27, 1% N2 supplement, 10 ng/ml FGF2, 10 ng/ml EGF, and 10 ng/ml PDGF-AA. After the formation of oligospheres, the OPCs were dissociated and plated onto PDL-coated dishes in growth medium (GM), as previously described (Wang et al., 2017). The cells were maintained for 5 days in OLG differentiation medium (DM) containing DMEM medium, 4 mM L-glutamine, 1 mM sodium pyruvate, 0.1% BSA, 50 μg/ml apotransferrin, 5 μg/ml insulin, 30 nM sodium selenite, 10 nM biotin, 10 nM hydrocortisone, 15 nM T3, 10 ng/ml CNTF, and 5 μg/ml NAC.

##### Rat hippocampal neurons

Hippocampal tissues were isolated from newborn SD rat, and digested by 0.25% trypsin solution containing DNase at 37°C for 30 min. The hippocampal cells were seeded onto PDL-coated coverslips (1 × 10^4^ cells/coverslip) in Neurobasal medium with 2% B27, 0.25% GlutaMAX^TM^ and 10% HS. After a period of 1 h, the medium was replaced by Neurobasal medium with 2% B27 and 0.25% GlutaMAX^TM^. Ara-C (1 mM) was added to the culture at day 4 to inhibit glia cell proliferation.

#### Lentivirus-Mediated shRNA Targeting Bcl11b

Previously, we found that the constructs of lentiviral vectors made in Biosettia (San Diego, CA, United States) were highly efficient in the inhibition of Bcl11b (NM_001277287) in rat glioma cells (Liao et al., 2016). Thus, we used the same lentiviral vector constructs for this study: pLV-mU6-EF1a-GFP-Puro-scramble (lenti-ctrl); and, pLV-mU6-EF1a-GFP-Puro-shBcl11b-916 (lenti-shBcl11b). The shRNA sequences are shown in **Table 2**. For gene transduction, GPCs/OPCs (1 × 10^6^ cells/dish) were seeded onto 60-mm petri dishes in GM, and lentiviruses (300 μl/dish) carrying shRNA against Bcl11b and scramble lentiviruses were separately added into the medium for 24 h. The shRNA genes were allowed to express for 48 h, and the transfectants were selected in the presence of puromycin (3 μg/ml), also for 48 h. The efficiency of the lentiviral particles for Bcl11b downregulation in the GPCs was confirmed by quantitative polymerase chain reaction (QPCR) and western blotting. GPCs infected by lenti-ctrl were referred to as ‘scramble,’ while cells infected by lenti-Bcl11b-KD were called ‘Bcl11b-KD’.

**Table 2:** Primer sequences for QPCR analysis and Sequences for shRNA against rat Bcl11b.

Gene	Sequence
Rat Bcl11b (NM“_001277287)	Forward (5′→3′): GCAGTCCAACCTAACCTGTGTC Reverse (5′→3′): GGGTGCCTTAATCAACCCTCAG
Rat CD133 (NM“_021751)	Forward (5′→3′): CCAGCGGCAGAAGCAGAACGA Reverse (5′→3′): GTCAGGAGAGCCCGCAAGTCT
Rat Sox2 (NM“_001109181)	Forward (5′→3′): CACAACTCGGAGATCAGCAA Reverse (5′→3′): CGGGGCCGGTATTTATAATC
Rat Bmi-1 (NM“_001107368)	Forward (5′→3′): GCGTTACTTGGAGACCAGCA Reverse (5′→3′): CTTTCCGATCCGACCTGCTT
Rat Hes1 (NM“_024360)	Forward (5′→3′): TACCCCAGCCAGTGTCAACA Reverse (5′→3′): TCCATGATAGGCTTTGATGACTTTC
Rat Hey1 (NM“_001191845)	Forward (5′→3′): AGCGCAGACGAGAATGGAAA Reverse (5′→3′): CGCTTCTCGATGATGCCTCT
Rat Hey2(NM“_130417)	Forward (5′→3′): CTTGACAGAAGTGGCGAGGTReverse (5′→3′): CATTGGGTTGGAGCAGGGAT
Rat p21(NM“_080782)	Forward (5′→3′): TGGACAGTGAGCAGTTGAGCReverse (5′→3′): ACACGCTCCCAGACGTAGTT
Rat Olig1(NM“_021770)	Forward (5′→3′): GAGGGGCCTCTTTCCTTGTCReverse (5′→3′): ACCGAGCTTCACAAGCCTAC
Rat Olig2(NM“_001100557)	Forward (5′→3′): GCTTAACAGAGACCCGAGCCReverse (5′→3′): GTGGCGATCTTGGAGAGCTT
Rat MBP(NM“_001025291)	Forward (5′→3′): GTGGGGGTAAGAGAAACGCAReverse (5′→3′): CGAACACTCCTGTGGAACGA
Rat PLP(NM“_030990)	Forward (5′→3′):GGCGACTACAAGACCACCATReverse (5′→3′):AATGACACACCCGCTCCAAA
Rat NFATc3(NM“_001108447)	Forward (5′→3′):TCTGACTTGGAACACCAGCCReverse (5′→3′): AAGCAGTCAGAGCAGTTGGT
Rat MOG(NM“_022668)	Forward (5′→3′): CCCAGCGCTTCAACATTACGReverse (5′→3′): GCACCTAGCTTGTTTGTGTCTG
Rat GAPDH(NM“_017008)	Forward (5′→3′): TCTACCCACGGCAAGTTCReverse (5′→3′): GATGTTAGCGGGATCTCG
Scramble shRNA	GCAGTTATCTGGAAGATCAGGTTGGATCCAACCTGATCTTCCAGATAACTGC
shBcl11b-916(NM“_001277287: nt637-656)	AAAAGAGCCTTCCAGCTACATTTGTTGGATCCAACAAATGTAGCTGGAAGGCTC

#### Quantitative Real-Time Polymerase Chain Reaction

The RNA (1 μg/sample) isolated from the GPCs and OPCs was reacted with M-MLV reverse transcriptase (Invitrogen) to generate cDNA, and then incubated with SYBR Green reagents (Roche) and specific primers (**Table 2**). The expression level of GAPDH was used as an internal control. StepOne Software v2.1 (Applied Biosystems) was used to determine the cycle-threshold (Ct) fluorescence values. The expression level of the target genes relative to the internal control was presented as 2^-ΔCT^, where ΔCT = (Ct_target_-Ct_GAPDH_).

#### Immunofluorescence

After harvesting, the cells were fixed in 4% paraformaldehyde for 10 min, and incubated in PBS containing 0.1% Triton-X100 for 30 min. The cultures then were incubated overnight at 4°C with primary antibodies (**Table 1**). To stain galactoceramide (GC), a major glycolipid of myelin, the cultures were directly incubated with anti-GC antibody after fixation with 4% paraformaldehyde, but without permeabilization by 0.1% Triton-X100. Alternatively, to carry out double immunofluorescence for NF200 (or GFP) and MBP (or PLP), the cultures were incubated with anti-NF200 (or anti-GFP) at 4°C overnight, and then with anti-MBP (or anti-PLP) at RT for 3 h. After reacting with the primary antibodies, appropriate secondary antibodies and FITC-avidin were added to the cultures at RT for 1 h and for 45 min, respectively. The immunostained cells were photographed under a Nikon E800 epifluorescence microscope equipped with a CCD camera and also under an Olympus FluoView laser scanning confocal microscope (FV1000, Japan).

#### Evaluation of Oligodendrocytic Differentiation

In addition to the morphological observations of OLG differentiation from GPCs or OPCs, OLG differentiation was evaluated by measurement of OLG cell-marker expression intensity, as described previously (Wang et al., 2017). MetaXpress software (Molecular Devices; Sunnyvale, CA, United States) and NIH ImageJ analysis software were used. Five randomly selected images (5–10 cells per image) were captured from each immunostained culture using the above-mentioned epifluorescence microscope with a 40X objective lens. The experiments were repeated in triplicate, and 70–100 cells in total per group were counted. The number of processes and branches per cell, the average length of the processes, and the total length of the outgrowing processes per cell in each field were quantified. The immunofluorescent intensity per cell and the number of immunostained cells in each field were also measured. The results are presented as the percentage of the data obtained from the Bcl11b-KD culture versus data from the scramble culture.

#### Western Blot Analysis

GPCs and OPCs were replated at a density of 1 × 10^6^ cells/60-mm onto petri dishes for various experiments. After harvesting, the total protein content (100 μg) was extracted from the cultures and lysis buffered in 1% NP-40, 1% Triton-X100, and 0.1% SDS, which it was loaded onto 10% SDS polyacrylamide gel. After electrophoresis, the protein was transferred to a nitrocellulose membrane and immunoblotted overnight at 4°C with primary antibodies (**Table 1**). The immunoblotted membrane was incubated with secondary antibodies conjugated with peroxidase for 60 min at RT. The signal was detected by chemiluminescence using the ECL-Plus detection system (PerkinElmer Life Sciences).

### Cell Growth Assays

MTT colorimetric assay, gliosphere formation assay, and colony formation assay were performed as described previously (Fang et al., 2014). GPCs and OPCs were maintained in GM either for different time periods (MTT assay), or for 7 days (gliosphere and colony formation assays). The cell proliferation of the GPCs in GM after 48 h was also examined using BrdU incorporation assay via the addition of BrdU (10 μM) into the culture 12 h before harvesting, following the previously described procedure (Wang et al., 2015). The number of gliospheres, colonies, and BrdU^+^ cells in the culture were counted using ImageJ analysis software (NIH, United States).

#### Chromatin Immunoprecipitation

A chromatin immunoprecipitation (ChIP) assay kit (Millipore) was employed, the experimental procedure for which was based on the manual provided by the vendor. Briefly, GPCs were seeded at a density of 1 × 10^7^ cells/100-mm petri dish for 2 days in GM. After 1% formaldehyde was added into the medium, the cells were removed and suspended in a cell-lysis buffer. After centrifugation, the resulting cell pellet was resuspended in a nuclear-lysis buffer for 10 min. The sample was then sonicated to produce DNA fragments at lengths of 200–600 bp, followed by incubation with anti-Bcl11b antibodies (or isotype IgG as negative control) and protein A/G magnetic beads at 4°C overnight. DNA-protein complexes were collected and treated by proteinase K at 62°C for 2 h. After DNA purification, the DNA fragments that potentially interacted with Bcl11b were analyzed by QPCR using specific primers (**Table 3**). Results from samples that had reacted with mouse isotype IgG (IgG) are referred to as negative control.

**Table 3:** Primer sequences for ChIP-QPCR analysis.

Promoter	Sequence
Rat *p21* (+2862 ∼ +2944)[Gene ID: 114851]	Forward (5′→3′): GCCCCTTTCTAGCTGTCTGGReverse (5′→3′): GCTCCTTCACCCATCCCTG
Rat *Olig1* (-1864 ∼ -1763)[Gene ID: 60394]	Forward (5′→3′): CGTACCGCTTATGTGCAGGGReverse (5′→3′): ACCCTACATTCCTAGCCATCG
Rat *Olig1* (-1487 ∼ -1264)[Gene ID: 60394]	Forward (5′→3′): CTGATAGCTGTGAGGGTGAAGReverse (5′→3′): CCCAGATGCTGGGAATACAA
Rat *Olig1* (-1121 ∼ -1030)[Gene ID: 60394]	Forward (5′→3′): TGAGCCAGCCACTAAAAGACAReverse (5′→3′): CTTCATCCTGGGGTGTCTGC
Rat *Olig1* (-575 ∼ -502)[Gene ID: 60394]	Forward (5′→3′): CAAAAGCTAACAAGTCCCGATCAReverse (5′→3′): CGCAGTTCAGTCGTTAAAACACC
Rat *Olig1* (-399 ∼ -261)[Gene ID: 60394]	Forward (5′→3′): CAGCTACAGCAGTTCCCAGTReverse (5′→3′): CTAGTTCAGCGGGTCATGCT
Rat *Olig1* (-240 ∼ -147)[Gene ID: 60394]	Forward (5′→3′): GCCCTATAAAGCTCCCTCCCReverse (5′→3′): CAGCCAGAGTTGCCAGAGAT
Rat *Plp* (-1521 ∼ -1423)[Gene ID: 24943]	Forward (5′→3′): GATCAGTGGGAGTGTGCAGGReverse (5′→3′): CACTCTCCCCTGTCCCCTAA
Rat *Plp* (-1173 ∼ -1065)[Gene ID: 24943]	Forward (5′→3′): AGTCCCAGAGATGCTCCTGAReverse (5′→3′): GAGGGGAATCAAGCAGCCAA
Rat *Plp* (-982 ∼ -862)[Gene ID: 24943]	Forward (5′→3′): GCTGCACTTTCGTAACAGGCReverse (5′→3′): AGGTAGTAGCTTCCCAGGGT
Rat *Plp* (-625 ∼ -433)[Gene ID: 24943]	Forward (5′→3′): TCTTGAGCCTGGTCACACACReverse (5′→3′): AGTTGGCCTTGACCATGGAA
Rat *Plp* (-368 ∼ -266)[Gene ID: 24943]	Forward (5′→3′): TCCTCACCAGGGCTACCATTReverse (5′→3′): AGGGGTCCTTAAATCCTCCCA
Rat *Plp* (-40 ∼ -109)[Gene ID: 24943]	Forward (5′→3′): TTTAAGGGGGTTGGCTGTCAReverse (5′→3′): AGTCTGTTTTGCGGCTGACT

#### Assessments of *in Vitro* Co-culture of GPCs and Neurons

After hippocampal neurons were cultured for 7 days, GPCs at the density of 1 × 10^4^ cells per coverslip were added into the hippocampal culture, and maintained in Neurobasal medium with 2% B27, 0.25% GlutaMAX^TM^ and T3 (30 ng/ml) for 7 days. The hippocampal neuron-OLG co-cultures were subjected to double immunofluorescence for NF200 and MBP. The assessments followed the methods described in our previous study (Wang et al., 2017). The intensity of MBP fluorescence, which overlapped with a neuronal fiber featuring immunoreactivity to NF200, was quantified using NIH ImageJ analysis software. Additionally, to further verify the overlap of the MBP^+^-OLG process with the NF200-immunostained fiber, the cultures were subjected to confocal imaging analysis to acquire a z-stack reconstructed from 7 sequential images at 1-μm intervals. 3D images, including x-z and y-z views, were obtained from the same z-stack to identify the overlapping regions of MBP- and NF200-immunostaining.

#### Cerebellar Slice Culture

The *ex vivo* cerebellar slice culture was modified and performed according to a previous study (Lee et al., 2015). Briefly, the rat sagittal-cerebellar slices at P7 were dissected at a thickness of 350 μm using a Microslicer^TM^ DTK-1000 vibratory tissue slicer. The tissue slices were then plated on Millicell-CM culture inserts (Millipore, 0.4 μm) and maintained on the surface of the slice culture medium (50% MEM with Earle’s salts, 35% Earle’s balanced salt solution, 15% heat-inactivated horse serum, 1% GlutaMAX^TM^) at 37°C for 9 days. The scramble and Bcl11b-KD GPCs were seeded onto a cerebellar slice at a number of 1 × 10^5^ cells/slice. After 48 h, the scramble GPCs/cerebellar slice and Bcl11b-KD GPCs/cerebellar slice cultures were fixed with 4% paraformaldehyde and permeabilized by 0.3% Triton X-100 in PBS, followed by immunofluorescence for MBP and NF200.

#### GPC Transplantation Followed by Lysolecithin Injection

Adult male SD rats (250 ± 30 g) were anesthetized by intraperitoneal injection of chloral hydrate (50 mg/kg) and placed in a stereotaxic frame (Stoelting). A midline incision was made and the underlying tissue removed using a scalpel. A hole was drilled in the exposed skull by a dentist drill fitted with a 0.9 mm diameter carbide dental burr at 2 mm to the right of the sagittal suture. A Hamilton syringe with a 25-gauge needle was inserted 2.5 mm into the brain (corpus callosum). The fluid (5 μl) containing 1% lysolecithin was slowly injected into the brain. After injection, the needle was maintained in place for 2 min to prevent leakage. At 3 days post injection (dpi), the hole was re-exposed, and 1 × 10^5^ GPCs in 5 μl PBS were injected into the brain at the same position. At 14 dpi, the rats were sacrificed and their brains removed. The brain tissues were fixed in 4% paraformaldehyde, and then cryoprotected in 30% (w/v) sucrose in PBS. The tissues were embedded in Tissue Tek OCT (Electron Microscopy Sciences), sectioned with a 15-μm thickness, and then subjected to immunofluorescence as described above.

#### Statistical Analysis

The statistical significance of the differences between the two groups of data was analyzed by a two-tailed unpaired Student’s *t*-test, with all data expressed as means ± SEM. In all comparisons, differences were statistically significant at *p* < 0.05.

## Results

### Reduced Stemness and Proliferation of Glial Progenitor Cells by Downregulation of Bcl11b Expression under the Growth Condition

The findings indicated that Bcl11b is highly expressed in mouse cortical neurons and hippocampal dentate gyrus granule neurons (Arlotta et al., 2005; Simon et al., 2012). Here, through immunofluorescence, we showed that Bcl11b expression was co-localized to CC1^+^-OLGs in the corpus callosum, as well as Olig2^+^-OLG lineage cells and NG2^+^-glial progenitor cells (GPCs) in the subventricular zone (SVZ) of the rat brain (Supplementary Figure S1). *In vitro* examination showed that Bcl11b was produced in A2B5^+^- and NG2^+^-rat GPCs (Supplementary Figure S2A). Bcl11b expression was also detectable in CNPase^+^-OLGs generated from rat GPCs (Supplementary Figure S2A). The mRNA levels of Bcl11b in neurons and distinct glial cells were also confirmed using primary cultures. Although Bcl11b showed considerable expression in neurons, OPCs were found to express only a moderate level of Bcl11b (Supplementary Figure S2B).

**FIGURE 1:**
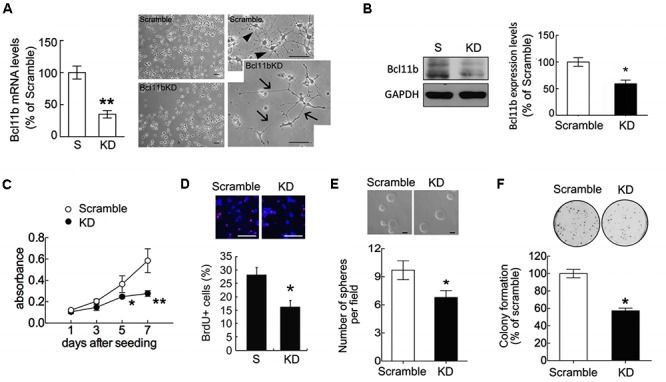
Reduction in GPC cell proliferation after Bcl11b gene downregulation. GPCs were infected by lenti-ctrl (scramble) and lenti-shBcl11b (Bcl11b-KD) as described in section “Materials and Methods.” The efficiency of Bcl11b downregulation in Bcl11b-KD GPCs was confirmed using QPCR for the measurement of Bcl11b mRNA expression **(A)** and western blot assay for Bcl11b protein production **(B)**. The phase-contrast images with low (left-panel) and high (right-panel) magnification were taken to examine the morphological change of GPCs after Bcl11b-KD **(A)**. Arrows in **(A)** indicate elongating processes extending from Bcl11b-KD GPCs compared to those of scramble GPCs (arrowheads). Scramble and Bcl11b-KD GPCs maintained in GM were subjected to MTT analysis **(C)**, BrdU incorporation assay **(D)**, sphere formation **(E)**, and colony formation assays **(F)**. Data are presented as means ± SEM of repeated independent experiments (*n* = 3–4). ^∗^*p* < 0.05, ^∗∗^*p* < 0.01 vs. scramble. Scale bar in **(A)** and **(D)** 50 μm; in **(E)** 100 μm.

To determine the function of Bcl11b in GPCs and OLGs, we performed lentivirus-mediated shRNA delivery against Bcl11b expression (Bcl11b-KD) in GPCs. Lenti-Bcl11b shRNA efficiently downregulated Bcl11b mRNA expression (**Figure 1A**) and protein production (**Figure 1B**) in GPCs compared to what we observed in GPCs infected by lenti-ctrl (scramble). In addition, it was observed that the morphology of the GPCs had altered into a shape with elongated processes after Bcl11b-shRNA transduction (**Figure 1A**, arrows). The examination of GPC cell growth at different times by MTT analysis or after 24 h by BrdU incorporation assay indicated that GPC cell growth was reduced in the GM after Bcl11b downregulation (**Figures 1C,D**). Moreover, the ability of GPCs to form glial spheres and colonies declined after Bcl11b-KD (**Figures 1E,F**).

**FIGURE 2:**
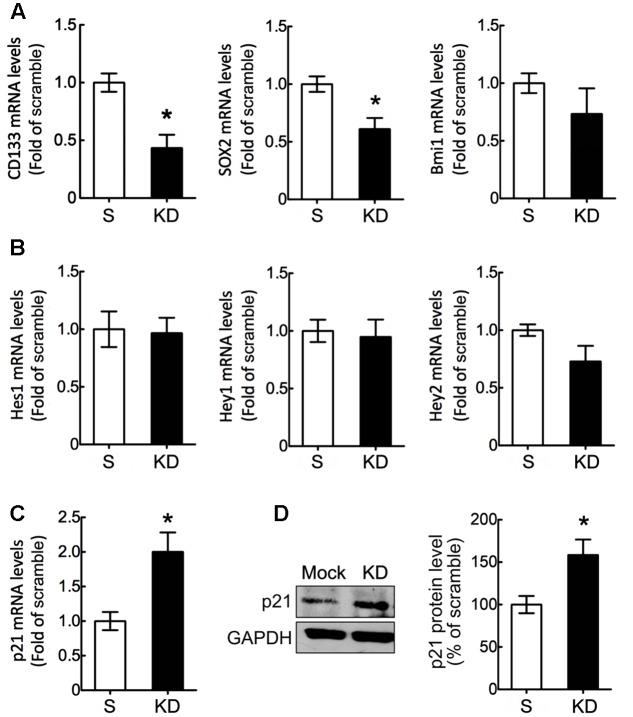
Downregulation of stemness-related genes in GPCs by Bcl11b knockdown. Scramble and Bcl11b-KD GPCs were maintained in GM for 48 h, and then subjected to QPCR analysis for the measurement of stemness-associated genes including CD133, Sox2, and Bmi1 **(A)**, as well as the effector genes (Hes1, Hey1, and Hey 2) of Notch signaling **(B)**. The expression of p21 mRNA in scramble and Bcl11b-KD GPCs was examined using QPCR **(C)**. Moreover, total proteins were extracted from scramble and Bcl11b-KD GPCs in GM for 48 h, and then subjected to western blotting **(D)** to measure p21 protein production. The intensity of immunoreactive bands corresponding to p21 and GAPDH as a loading control was quantified by ImageJ software. Data shown in **(A–D)** are presented as means ± SEM of the three independent experiments. ^∗^*p* < 0.05 vs. the scramble.

Based on our previous findings that Bcl11b-KD reduced the expression of stemness-related genes (Sox2 and Bmi1) in glioma cells (Liao et al., 2016), we also examined the expression of stemness-related genes in the scramble and Bcl11b-KD cultures. As shown in **Figure 2**, the expression of Sox2 – but not of Bmi1 – was reduced in Bcl11b-KD GPCs compared to that detected in the scramble culture. Moreover, we found that CD133, a stem-cell marker, was downregulated in the GPCs after Bcl11b-KD (**Figure 2A**). These data reveal that Bcl11b actively participates in the regulation of GPC cell proliferation. Findings in a separate study indicated that Bcl11b can trigger early differentiation of epidermal keratinocytes by binding to the Notch1 promoter to promote the expression of Notch1 (Zhang et al., 2012). Thus, we examined the expression of Notch downstream genes (Hes1, Hey1, and Hey2) in the GPCs cultured in GM for 48 h. The results indicated that the change in the expression of the three genes was insignificant in both the scramble and Bcl11b-KD GPCs (**Figure 2B**).

Given that Bcl11b can bind to the Sp1 promoter binding site of the cell cycle repressor p21 to cause p21 gene silencing (Cherrier et al., 2009), the p21 mRNA expression in Bcl11b-KD GPCs was compared to that detected in the scramble GPCs. The results showed that p21 mRNA expression and its protein production were significantly increased in the GPCs after Bcl11b-KD (**Figures 2C,D**). These results suggest that the reduced cell proliferation of GPCs in GM might be due to the increased effect of p21 after Bcl11b downregulation.

### Upregulation of OLG-Specific Gene Expression in GPCs after Bcl11b Gene Knockdown

**FIGURE 3:**
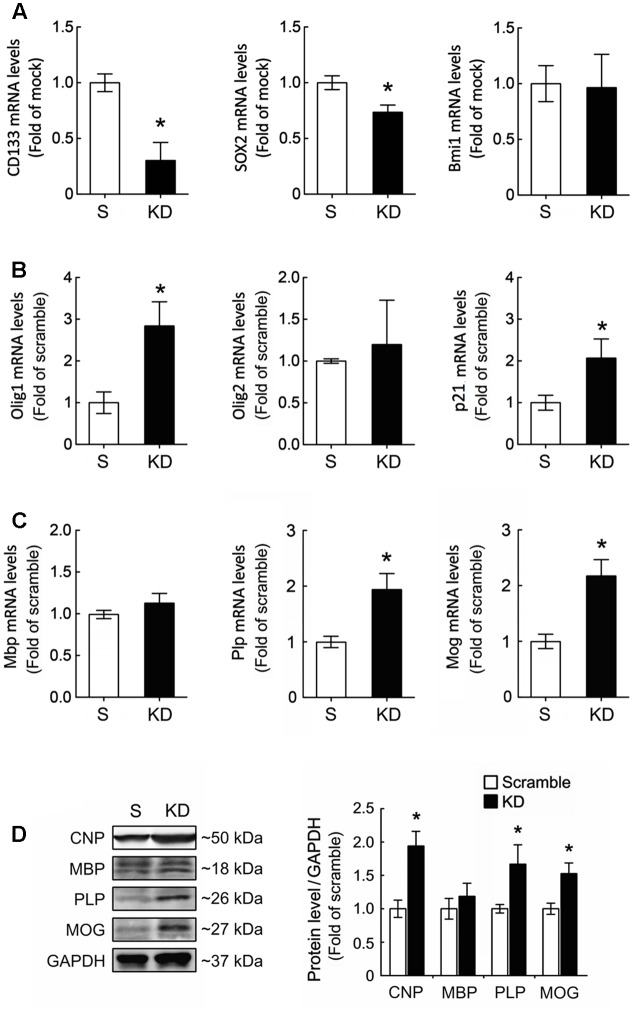
Upregulation of Olig1 and p21 expression in GPCs after Bcl11b gene downregulation. **(A)** Scramble and Bcl11b-KD GPCs were cultured in DM for 48 h, and then subjected to QPCR analysis for the measurement of stemness-associated genes, CD133, Sox2, and Bmi1. **(B,C)** Moreover, transcription factor-associated OLG differentiation (i.e., Olig1 and Olig2), p21, and myelin-related genes were examined by QPCR analysis. **(D)** In addition, total proteins were extracted from scramble and Bcl11b-KD GPCs cultured in DM for 5 days, and then subjected to western blot analysis for the examination of OLG cell markers, CNPase, MBP, PLP, and MOG. The intensity of immunoreactive bands shown in the right panel was quantified by ImageJ software, and normalized by the level of GAPDH that is as a loading control. Data are presented as means ± SEM of the three independent experiments. ^∗^*p* < 0.05 vs. the scramble.

Our findings, as indicated above, raise the question of whether Bcl11b-KD induced the cell death of GPCs or instructed the GPCs toward the differentiation of OLGs. Since the cell death of GPCs with Bcl11b-KD in GM or in DM was not observed (data not shown), we next evaluated the differentiation ability of GPCs after Bcl11b-KD. When GPCs were cultured in DM for 48 h to induce OLG differentiation, the expression of stemness-related genes (i.e., CD133 and Sox2) was significantly reduced compared to the scramble culture (**Figure 3A**). No change was observed in Bmi1 expression after Bcl11b-KD. Examination of Olig1 and Olig2, two critical bHLH transcription factors specific for OLG differentiation, indicated that Bcl11b-KD caused the upregulation of Olig1, but not of Olig2 (**Figure 3B**). This also provides evidence that Bcl11b-KD can promote OLG lineage specification. Moreover, an increase in p21 gene expression was observed when Bcl11b-KD GPCs were cultured in DM (**Figure 3B**). Furthermore, our results showed that Bcl11b-KD induced upregulated gene and protein expression of myelin proteolipid protein (PLP) and myelin oligodendrocyte glycoprotein (MOG), which are myelin proteins in mature OLGs (**Figures 3C,D**). However, MBP mRNA and protein expression was not affected by Bcl11b-KD (**Figures 3C,D**). In addition, the protein level of CNPase, a myelin-associated enzyme and a marker for OLG differentiation, was upregulated in the Bcl11b-KD culture (**Figure 3D**). These results reveal that Bcl11b-KD in the GPCs increased the expression of crucial proteins associated with the progression of the OLG lineage.

### Interaction of Bcl11b with the Promoter Regions of Olig1 and PLP

**FIGURE 4:**
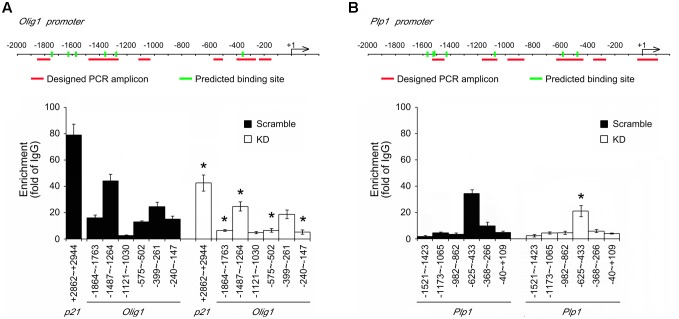
Identification of potential Bcl11b binding regions located in Olig1 and Plp1 promoters. ChIP assay was conducted as described in Section “Materials and Methods” to verify the predicted Bcl11b binding sites located at the promoter regions of Olig1, Plp1 and p21. The predicted Bcl11b binding sites are labeled as green rods. The primers that match to the flanking regions indicated by red lines in Olig1 **(A)** and Plp1 **(B)** promoters were designed and synthesized. ChIP products prepared from scramble and Bcl11b-KD GPCs were analyzed by QPCR using the synthesized primers. The data shown in **(A,B)** are normalized over those obtained from IgG samples. Interaction of Bcl11b and p21 promoter is referred to be a positive control. Data are presented as means ± SEM of the four independent experiments. ^∗^*p* < 0.05 vs. the scramble.

It has been reported that Bcl11b is associated with the Sp1 binding sites containing the p21 promoter, and acts as a repressor for p21 expression (Cherrier et al., 2009). Through ChIP analysis, we verified that Bcl11b can bind to the Sp1 sequence located at the p21 promoter in the scramble GPCs (**Figure 4**). This finding reflects our observations of an increased expression of p21 in the GPCs after Bcl11b-KD (**Figure 2**). Through the ChIP method combined with high-throughput sequencing, the potential DNA binding sequences of Bcl11b have been identified (Tang et al., 2011). Accordingly, we used a nucleotide blast search for these potential Bcl11b binding sites on the Olig1 and Plp1 promoters, for which 6 and 7 potential segments were predicated for Bcl11b binding to within 2000 nt upstream sequences of the Olig1 and Plp1 promoters, respectively (**Figure 4**). To examine if Bcl11b can interact with the promoter of Olig1, ChIP experiments were performed using 6 designed primer pairs for the Bcl11b binding sites at the Olig1 promoter in both the scramble and Bcl11b-KD cultures (**Figure 4A**). The ChIP assay using anti-Bcl11b in combination with QPCR indicated that Bcl11b had a strong interaction with the segment (-1487 ∼-1264 bp) encompassing two predicted Bcl11b binding sites located at the Olig1 promoter (**Figure 4A**). This interaction, however, declined in Bcl11b-KD GPCs. In addition, Bcl11b was able to bind to the sequence at -625 ∼-433 bp of the Plp1 promoter in both cultures (**Figure 4B**), despite reduced binding being observed in Bcl11b-KD GPCs. Taken together, these findings, in conjunction with the results that Bcl11b-KD caused the upregulation of Olig1 and PLP in the GPCs, suggest that Bcl11b might act as a transcriptional repressor of these genes.

### Enhanced Differentiation of GPCs toward OLGs by the Downregulation of Bcl11b Gene Expression

**FIGURE 5:**
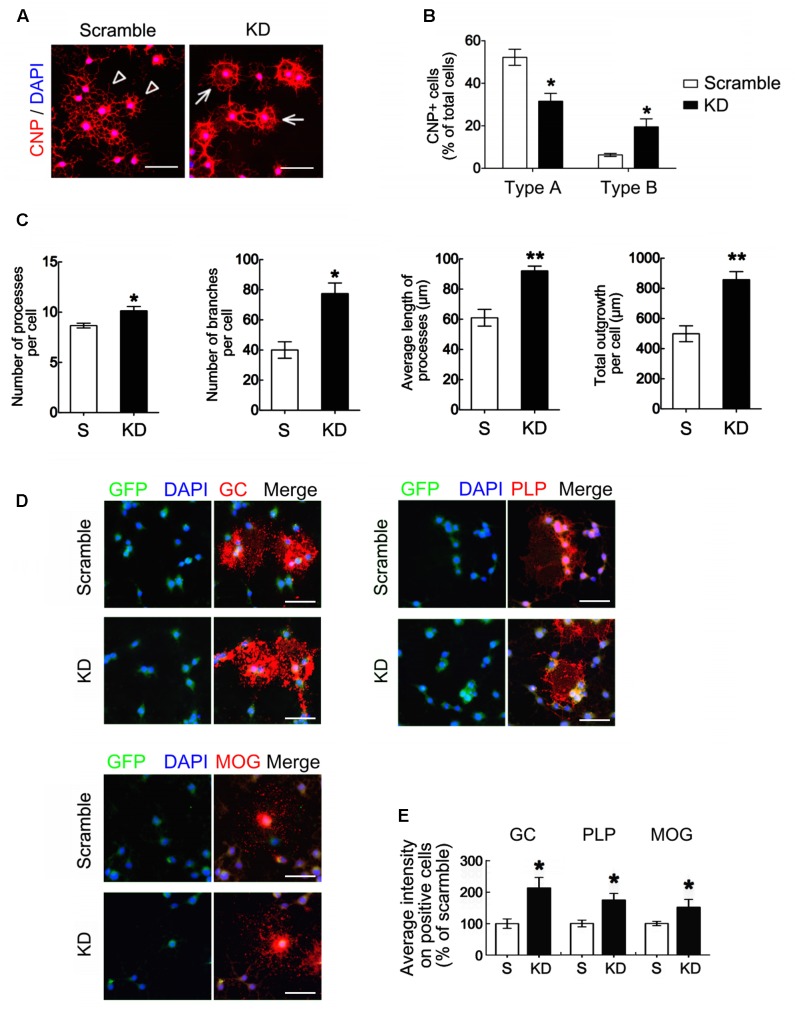
Oligodendrocyte differentiation and maturation enhanced by Bcl11b gene knockdown. **(A,B)** Scramble and Bcl11b-KD GPCs were seeded and maintained in DM for 5 days. The cultures were subjected to immunofluorescence using anti-CNPase antibody **(A)**. Arrowheads indicate the differentiated cells with branching processes (type A). Arrows point to the cells extending the processes to form a ring-shaped network (type B). CNPase^+^-Type A and CNPase^+^-Type B cells in scramble and Bcl11b-KD cultures were quantified using ImageJ software **(B)**. **(C)** The images taken from scramble and Bcl11b-KD cultures were further analyzed by MetaXpress software to measure the number of cell processes per cell, the branching numbers per cell, the average length per process, and the total process length per cell. **(D,E)** Scramble and Bcl11b-KD cultures were subjected to double immunofluorescence for GC, PLP, and MOG, combined with GFP as well as DAPI nuclear counterstaining **(D)**. The immunofluorescent intensity of each immunoreactive cell in the cultures was measured using ImageJ software **(E)**. Data are presented as means ± SEM of at least three independent experiments (*n* = 70–100 cells per group). ^∗^*p* < 0.05 vs. the scramble. Scale bar in **(A,D)**, 50 μm.

We used immunofluorescence to compare the morphological differences between the OLGs generated from the scramble GPCs and those from the Bcl11b-KD GPCs. The cultures were maintained in DM for 5 days to stimulate the differentiation of OLGs from GPCs. Through CNPase immunostaining, we observed that the OLGs generated from the scramble GPCs and those from the Bcl11b-KD GPCs had a shape with either multi-polar interconnected fine processes, termed as Type A (**Figure 5A**, arrowheads), or formed a ring-like structure of complex interwoven thick processes, named as Type B (**Figure 5A**, arrows). The process thickness was 1.60 ± 0.13 μm for Type A, and 3.10 ± 0.31 μm for Type B (Type A vs. Type B, *p* < 0.001). The number of CNPase^+^-OLGs with the Type B shape was significantly higher in the Bcl11b-KD culture than in the scramble culture (**Figure 5B**). These results indicate that Bcl11b-KD changed the morphology of the OLGs derived from GPCs toward a more complex and mature shape. In addition, Bcl11b-KD increased the number of OLG processes and process branches per cell (**Figure 5C**). Increases in the average length of the cell processes and in the total process length per cell were observed (**Figure 5C**). In addition, a stronger GC immunoreactivity was observed in the OLGs derived from the Bcl11b-KD GPCs (**Figures 5D,E**). MOG and PLP immunofluorescence also showed enhanced differentiation of Bcl11b-KD GPCs toward mature OLGs when these cells were cultured in DM over 5 days.

**FIGURE 6:**
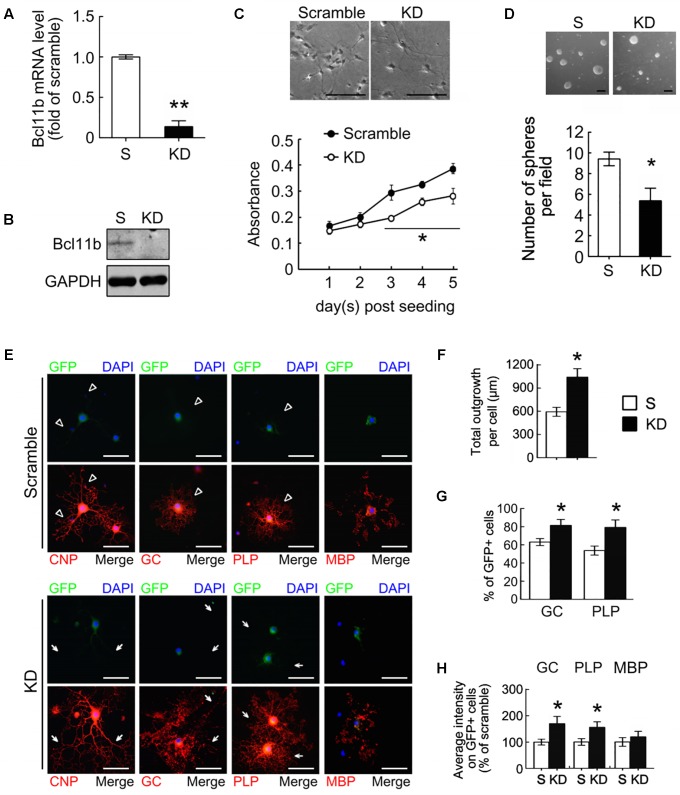
Oligodendrocyte differentiation from OPCs promoted by Bcl11b gene knockdown. OPCs were derived from primary NSCs as described in Materials and Methods. OPCs were infected by lenti-ctrl (scramble) and lenti-sh-Bcl11b (Bcl11b-KD) and maintained in GM with puromycin for 2 days. The efficiency of Bcl11b-KD was measured by QPCR **(A)** and western blot **(B)**. The scramble- and Bcl11b-KD OPCs maintained in GM were subjected to MTT analysis **(C)**, and the sphere formation assay **(D)**. In addition, the scramble- and Bcl11b-KD OPCs were cultured in DM to generate OLGs for 5 days, followed by immunofluorescence for CNPase, GC, PLP, and MBP **(E)**. The total length of CNPase^+^-cell processes per OLG cultures was analyzed **(F)**. Moreover, the percentage of GC^+^-OLGs and PLP^+^-OLGs in the scramble and Bcl11b-KD was measured **(G)**. Moreover, the intensity of immunofluorescence for GC, PLP, and MBP in each cell was measured by ImageJ software. The results show the average fluorescence intensity of Bcl11b-KD cells over those of scramble cells **(H)**. Data are presented as means ± SEM of at least three independent experiments (*n* = 70–100 cells per group for **F,G**). ^∗^*p* < 0.05 vs. the scramble. Scale bar in **(C,D)**, 100 μm; in **(E)**, 50 μm.

We also used neural stem cells (NSCs) prepared from rat cortical tissues at E14 to generate OPCs. The efficiency of Bcl11b-KD in the OPCs by lentiviruses was examined (**Figures 6A,B**). Note that there was approximately 95% PDGFRα^+^-cells in the scramble and Bcl11b-KD cultures maintained in GM (Supplementary Figure S3A). Bcl11b-KD causes decreased cell growth and sphere formation of the OPCs, which is comparable with the results from the GPCs (**Figures 6C,D**). Moreover, the findings were verified by Ki67 immunostaining, which showed lower amount of Ki67^+^/GFP^+^-cells in the Bcl11b-KD culture than that seen in the scramble culture (Supplementary Figure S3B). The morphology of CNPase^+^-OLGs differentiated from the scramble NSCs is shown in **Figure 6E** (arrowheads). In comparison, the CNPase^+^-OLGs generated from the NSCs that received Bcl11b-KD, showed elongated processes with a complex structure (**Figure 6E**, arrows). The total outgrowth length per CNPase^+^-OLG from the Bcl11b-KD NSCs was greater than that of the CNPase^+^-OLGs generated from the scramble culture (**Figure 6F**). In addition, the GC^+^-OLGs had visible cell processes extending from their cell bodies (**Figure 6E**, arrows), and were more numerous in the Bcl11b-KD culture than in the scramble culture (**Figure 6F**). Increased GC immunoreactivity and GC^+^-cell number in the Bcl11b-KD cultures were also observed (**Figures 6G,H**). Moreover, PLP^+^-OLGs derived from Bcl11b-KD NSCs displayed a cell form with more complex processes (**Figure 6E**, arrows) compared to those observed in the scramble culture (**Figure 6E**, arrowheads). Furthermore, although an increase in PLP immunoreactivity and the number of PLP^+^ cells was detected in the Bcl11b-KD culture (**Figures 6G,H**), Bcl11b-KD did not change the expression of MBP. These results demonstrate that the downregulation of Bcl11b gene expression in GPCs and NSCs caused an upregulation of GC and PLP, and promoted the differentiation of premyelinating OLGs.

**FIGURE 7:**
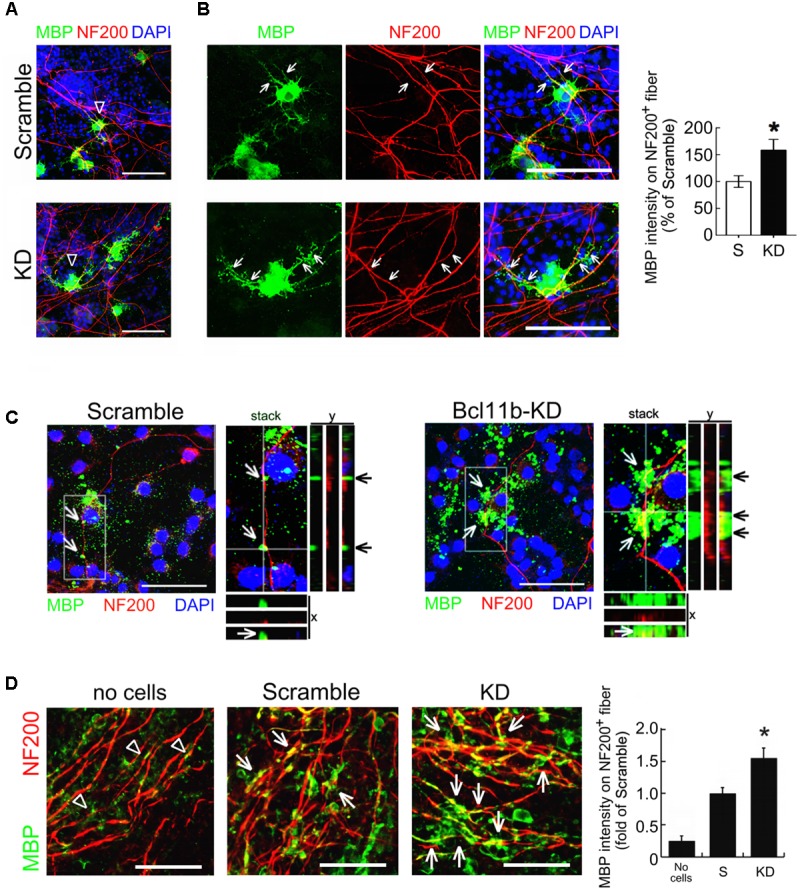
Increased premyelinating processes of Bcl11b-KD GPCs along neuronal fibers. **(A)** Hippocampal neurons were co-cultured for 7 days with scramble and Bcl11b-KD GPCs. The cultures were then subjected to double immunofluorescence using anti-NF200 (red) and anti-MBP (green) antibodies. **(B)** The images with a higher magnification display the representative regions indicated by arrowheads in **(A)**. Arrows indicate the hippocampal fibers overlapping with MBP^+^- processes extending from scramble OLGs and Bcl11b-KD OLGs, respectively. The intensity of MBP immunostaining overlapping to NF200^+^- hippocampal fibers (arrowheads and arrows) in the scramble and Bcl11b-KD culture was quantified. **(C)** 3D-confocal imaging analysis was conducted to show the overlapping of MBP^+^-OLG processes (green) to NF200^+^-neuronal fibers (red). Arrows indicate the MBP immunostaining co-localized with NF200^+^-regions in the longitudinal *y-z* and transverse x–z views from a z-stack image. **(D)** Scramble and Bcl11b-KD GPCs were seeded onto rat cerebellar slice culture for 7 days. The cultures were then subjected to double immunofluorescence for MBP (green) and NF200 (red). The immunoreactive intensity of MBP overlapping to NF200^+^ fiber (arrows) was quantified by ImageJ software (right panel). Note that MBP^+^-cell debris was observed in the culture without the addition of GPCs (arrowheads). Data are presented as means ± SEM of three independent experiments (*n* = 20–30 cells per group). ^∗^*p* < 0.05 vs. scramble. Scale bar, 50 μm.

A co-culture of GPCs with rat hippocampal neurons was established to further evaluate the effect of Bcl11b on OLG maturation. The co-culture was incubated for 7 days, and then subjected to double immunofluorescence for the identification of OLGs and neurons using anti-MBP and anti-NF200, respectively. The results displayed NF200^+^-neuronal fibers covered by MBP^+^-cell processes extending from the Bcl11b-KD OLGs (**Figures 7A,B**, arrows). By comparison, NF200^+^-neuronal fibers were not covered to the same degree by the scramble OLGs (**Figures 7A,B**, arrows). Quantitative analysis also showed that the intensity of the MBP immunofluorescence overlapping onto the NF200^+^-neuronal fibers in the Bcl11b-KD co-culture was higher than that in the scramble co-culture (**Figure 7B**). The 3D reconstruction imaging (xy, xz, and yz planes) from the serial confocal images further verified the intensive MBP immunoreactivity overlaid onto the NF200^+^-neuronal fibers in the Bcl11b-KD co-culture (**Figure 7C**, arrows). In contrast, weak MBP immunofluorescence spotted around the NF200^+^-neuronal fibers was seen in the scramble GPC/neuronal co-culture (**Figure 7C**, arrows). Moreover, the Bcl11b-KD GPCs were seeded onto *ex vivo* cerebellar-slice cultures for further examination. The Bcl11b-KD GPC/cerebellar tissue-slice co-culture displayed more MBP^+^ processes over NF200^+^ fibers compared to that in the scramble culture (**Figure 7D**). These observations further confirm that Bcl11b downregulation can progress OLG maturation.

### Differentiation of Bcl11b-KD OLGs*in Vivo*

**FIGURE 8:**
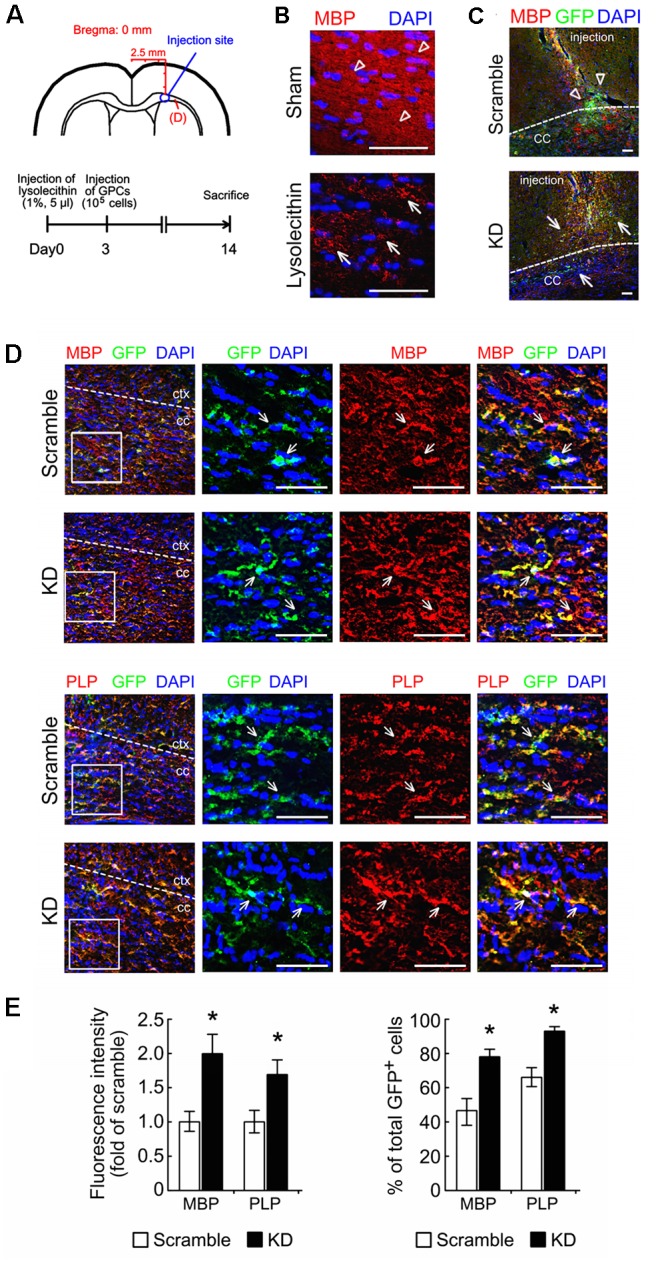
Improved differentiation of OLGs derived from Bcl11b-KD GPCs under a demyelinating condition. **(A)** The diagrams show the injection site of lysolecithin and GPCs just above the corpus callosum (CC, upper panel), and indicate the time points (lower panel) for the injection of 1% lysolecithin at Day 0 and GPCs (scramble and Bcl11b-KD GPCs) at Day 3. The animals were sacrificed at Day 14 after lysolecithin injection to investigate the differentiation of implanted GPCs into OLGs at the demyelinating CC regions labeled by **(D)**. **(B)** The brain sections that were prepared from animals having received lysolecithin injection were subjected to MBP immunostaining (red) and DAPI nuclear counterstaining (blue). The sham-operated group was only receiving vehicle. MBP^+^ debris was clearly observed in lysolecithin-injected CC (arrows). Diffuse and dense MBP staining was seen in the sham-operated group. **(C)** The brain sections receiving lysolecithin injection were subjected to double immunofluorescence for GFP (green) and MBP (red) to identify OLGs derived from implanted GPCs at the injection site. **(D)** By double immunofluorescence, GFP^+^/MBP^+^-OLGs (upper panel) and GFP^+^/PLP^+^-OLGs (lower panel) derived from implanted scramble and Bcl11b-KD GPCs in the CC adjacent to the injection site **(A)** were indicated by arrows. **(E)** To examine OLGs derived from implanted GPCs in the CC, the immunoreactive intensity of MBP and PLP in GFP^+^-cells shown in **(D)** was measured by ImageJ software. The results are shown as the fold of the scramble group. The results are shown as the percentage of double immunostained OLGs (GFP^+^/MBP^+^, GFP^+^/PLP^+^) over the total number of GFP^+^-cells in the field. Data in **(E)** are presented as means ± SEM from at least 3 animals in each group. The dash lines shown in **(C)** designate the border between cortex and CC. ^∗^*p* < 0.05 vs. scramble. Scale bar, 50 μm.

An *in vivo* model of focal demyelination that was induced by the injection of lysolecithin into the corpus callosum of adult rats was performed (**Figure 8A**). In this model, demyelination was detected at 3 dpi through examination of MBP^+^-debris at the injection site (**Figure 8B**, arrows), while the dense MBP immunoreactivity remained in the corpus callosum receiving-vehicle injection (**Figure 8B**, arrowheads). To further examine the differentiation of GPCs at the demyelinated site, the scramble and Bcl11b-KD GPCs were separately implanted into the corpus callosum 3 days after the lysolecithin injection (**Figure 8C**). OLGs derived from the implanted GPCs were identified by double immunofluorescence for GFP and MBP. The GFP^+^-scramble cells were found primarily at the injection site at 11 dpi (**Figure 8C**, arrowheads), whereas some of GFP^+^/MBP^+^-cells were found around the injection site in the Bcl11b-KD group (**Figure 8C**, arrows). The cell processes with intense MBP and PLP immunostaining were further detected in the GFP^+^ Bcl11b-KD GPCs in the corpus callosum (**Figure 8D**, arrows). In contrast, the GFP^+^-scramble cells expressed less MBP immunostaining (**Figure 8D**, arrowheads). The intensity of MBP and PLP immunostaining in the GFP^+^-OLGs in the corpus callosum was higher when receiving Bcl11b-KD GPCs than with the scramble group (**Figure 8E**). In addition, the proportion of MBP and PLP expressing cells with respect to GFP^+^-OLGs was also increased by Bcl11b-KD (**Figure 8E**). Notably, the amount of CC1^+^/GFP^+^-OLGs derived from Bcl11b-KD GPCs was higher than that observed in the scramble group (Supplementary Figures S4A,C), although the total amount of GFP^+^-cells at the adjacent area to the injection site in the Bcl11b-KD group was lower than that analyzed in the scramble group (Supplementary Figure S4B), These results demonstrate that Bcl11b-KD GPCs effectively generate mature OLGs in the demyelinating region of the brain.

## Discussion

Here, we show that Bcl11b expression can be detected in the GPCs within the SVZ of rat and mouse brains (Supplementary Figure S1). Moreover, Bcl11b expression declines in OLGs generated from OPCs. Further the *in vitro* study demonstrated that the downregulation of Bcl11b in GPCs caused the upregulation of the cell cycle inhibitor p21, and increased premyelinating OLGs generated from the GPCs. In addition, we report that Bcl11b might function as a transcriptional repressor for Olig1 and PLP to downregulate their gene expression in GPCs. The observations from the co-culture system of GPCs with hippocampal neurons and with the *ex vivo* cerebellar slice cultures also indicated that MBP expression can be upregulated in OLGs derived from Bcl11b-KD GPCs. The *in vivo* study using a chemically induced demyelinating animal model indicated that Bcl11b downregulation can promote implanted GPCs to differentiate into OLGs in the demyelinating region. These findings demonstrate the positive role of Bcl11b in the cell growth of GPCs and OPCs, but point to its negative effect on the progression of the OLG lineage to maturation.

Bcl11b has been reported to act as a transcriptional repressor to silence the expression of p21 (Cherrier et al., 2009). Our previous study using human and rat glioma cells also showed that p21 expression was upregulated after the downregulation of Bcl11b expression in glioma cell lines, suggesting that Bcl11b is an important regulator for glioma cell expansion through the repression of p21 action (Liao et al., 2016). The results from the present study showing that the proliferation of GPCs in GM was suppressed after Bcl11b-KD along with the upregulation of p21, also indicate that Bcl11b is involved in the maintenance of GPC stemness via the repression of p21 expression. Given that the ablation of p21 gene expression is known to inhibit OLG differentiation (Zezula et al., 2001), the upregulation of p21 after Bcl11b-KD might be involved in the differentiation of GPCs into OLGs. In addition, although Sox2 as a transcription factor is required for the self-renewal of OPCs, it blocks OLG differentiation (Zhao et al., 2015). Our observations that Bcl11b-KD attenuated the expression of Sox2 in GPCs under the condition of GM or DM, raise the possibility that the downregulation of Bcl11b might facilitate OLG differentiation from GPCs. Indeed, the expression of OLG markers (CNPase, PLP, and MOG) can be increased in Bcl11b-KD GPCs or Bcl11b-KD OPCs, demonstrating that a decline in Bcl11b expression can promote GPC differentiation into mature OLGs.

It has been reported that neuronal progenitor proliferation and post-mitotic neuron differentiation in the hippocampus declined in Bcl11b mutant mice during development (Simon et al., 2012). In addition, the deficiency of Bcl11b can cause postmitotic vomeronasal sensory neurons to selectively undergo cell apoptosis (Enomoto et al., 2011). A lack of Bcl11b functionality has been reported to cause inefficient differentiation of spiny neurons in striatal medium, with reduced expression of mature striatal markers (Arlotta et al., 2008). Here, we show that the downregulation of Bcl11b expression in GPCs and OPCs can reduce their proliferation under the growth condition, but promote the OLG lineage progression. Our results, in conjunction with findings on neuronal development, demonstrate that functional Bcl11b is required for neural progenitor cell proliferation. Despite the expression of Bcl11b in rat and mouse OLGs located in the white matter being different, it remains to be further investigated if OLG differentiation could be impaired in the absence of functional Bcl11b.

Several Bcl11b binding sites have been assumed to occur at the promoters of striatal genes (Desplats et al., 2008). It has also been reported that the expression of Olig1, one of the key transcription factors for OLG differentiation, was induced in Bcl11b-deficient neurons (Enomoto et al., 2011). These findings, together with our observations of an increased expression of Olig1 in OLGs derived from Bcl11b-KD GPCs, raise the possibility that Bcl11b might be involved in the regulation of OLG-specific gene expression. By searching a transcription factor prediction database for potential Bcl11b binding sites in 2-kb long promoter regions of rat OLG-specific genes (Olig1, Olig2, MBP, and PLP), we validated six and seven (predicted) Bcl11b binding sites located at the Olig1 and PLP promoters, respectively. Our results from the ChIP assay further confirm that Bcl11b exhibits high binding affinity to the specific promoter regions of Olig1 (-1487 ∼-1264) and PLP (-625 ∼ -433). Although the 2-kb long promoter regions of MBP contain 3 predicted binding sites with Bcl11b, the binding affinity of Bcl11b derived from the MBP promoters was extremely weak (data not shown). This is in accordance with our observation that MBP transcription was not affected by Bcl11b-KD. In other words, Bcl11b most likely acts as a transcriptional repressor of Olig1 and PLP, but not of MBP. Yet, the regulation of these genes by Bcl11b could also be due to the action of Bcl11b-induced chromatin remodeling via its interaction with nucleosome remodeling as well as histone deacetylase (NuRD) complex and SWI/SNF complex, which has been found in the control of T cells and neural development (Cismasiu et al., 2005; Topark-Ngarm et al., 2006; Son and Crabtree, 2014). Taken together, Bcl11b potentially deters the differentiation of GPCs into OLGs through the repression of Olig1 and PLP. Accordingly, the reduction of Bcl11b can decrease its inhibition in the expression of these two molecules, and subsequently promote OLG differentiation and maturation. Nevertheless, the upstream regulators that can mediate Bcl11b expression during OLG development remain to be determined.

Proteolipid protein is not only required for the stabilization of the myelin membrane and axonal structure (Uschkureit et al., 2000), but is also involved in the transportation of myelin membranes in the OLG secretory pathway (Nave and Werner, 2014). Thus, the upregulation of PLP expression in OLGs derived from Bcl11b-KD GPCs could promote the maintenance of OLG interaction with neuronal fibers in hippocampal neuron co-cultures and cerebellar-slice systems, as well as in chemically induced demyelination of the corpus callosum. Sox10 can directly induce MBP expression (Liu et al., 2007), or cooperate with Olig1 to increase MBP expression (Li et al., 2007). However, based on our *in vitro* study, we found no change in the Sox10 gene expression after Bcl11b-KD. This may explain why MBP gene expression and its protein production did not increase in our *in vitro* study. Yet, MBP expression has been known to be upregulated in the presence of neurons and neuronal factors (Bologa et al., 1986; Jensen et al., 2000; Simons and Trajkovic, 2006). Here, we provide important evidence that MBP expression in OLGs derived from Bcl11b-KD GPCs was increased under the condition of hippocampal neuron co-culture and cerebellar-slice culture. In addition, our *in vivo* results demonstrate that an increase in MBP immunoreactivity was detected in OLGs derived from implanted Bcl11b-KD GPCs toward the demyelination of white matter regions. These *ex vivo* and *in vivo* results further revealed that the downregulation of Bcl11b expression can facilitate glial progenitors to induce OLG maturation. Moreover, in response to neuronal signals, OLGs derived from GPCs, in tandem with the downregulation of Bcl11b expression, could possibly foster myelination.

## Conclusion

Bcl11b is essential for maintaining the proliferation of glial progenitors and their stemness properties. Reduced expression of Bcl11b in glial progenitors can contribute to the differentiation of GPCs toward mature OLGs. Moreover, our *in vitro* and *in vivo* findings provide important information for the development of an effective cell therapeutic strategy for demyelinating disorders via the downregulation of Bcl11b expression.

## Author Contributions

Study concept and design: C-YW and S-FT. Acquisition of data: C-YW, K-MF, and C-HH. Analysis and interpretation of data: C-YW, Y-TS, and S-FT. Drafting of the manuscript: C-YW, Y-TS, and S-FT. Critical revision of the article for important intellectual content: C-YW and S-FT. Statistical analysis: C-YW and K-MF. Obtained funding: C-SY and S-FT. Technical and material support: C-SY and S-FT.

## Conflict of Interest Statement

The authors declare that the research was conducted in the absence of any commercial or financial relationships that could be construed as a potential conflict of interest.
