# Supplementary material for: Function of B-Cell CLL/Lymphoma 11B in Glial Progenitor Proliferation and Oligodendrocyte Maturation
Source: Front Mol Neurosci. 2018 Jan 24;11:4. doi: 10.3389/fnmol.2018.00004 (PMC5787563; doi:10.3389/fnmol.2018.00004)
Supplement: Supplementary file 1 [file Presentation_1.PDF]

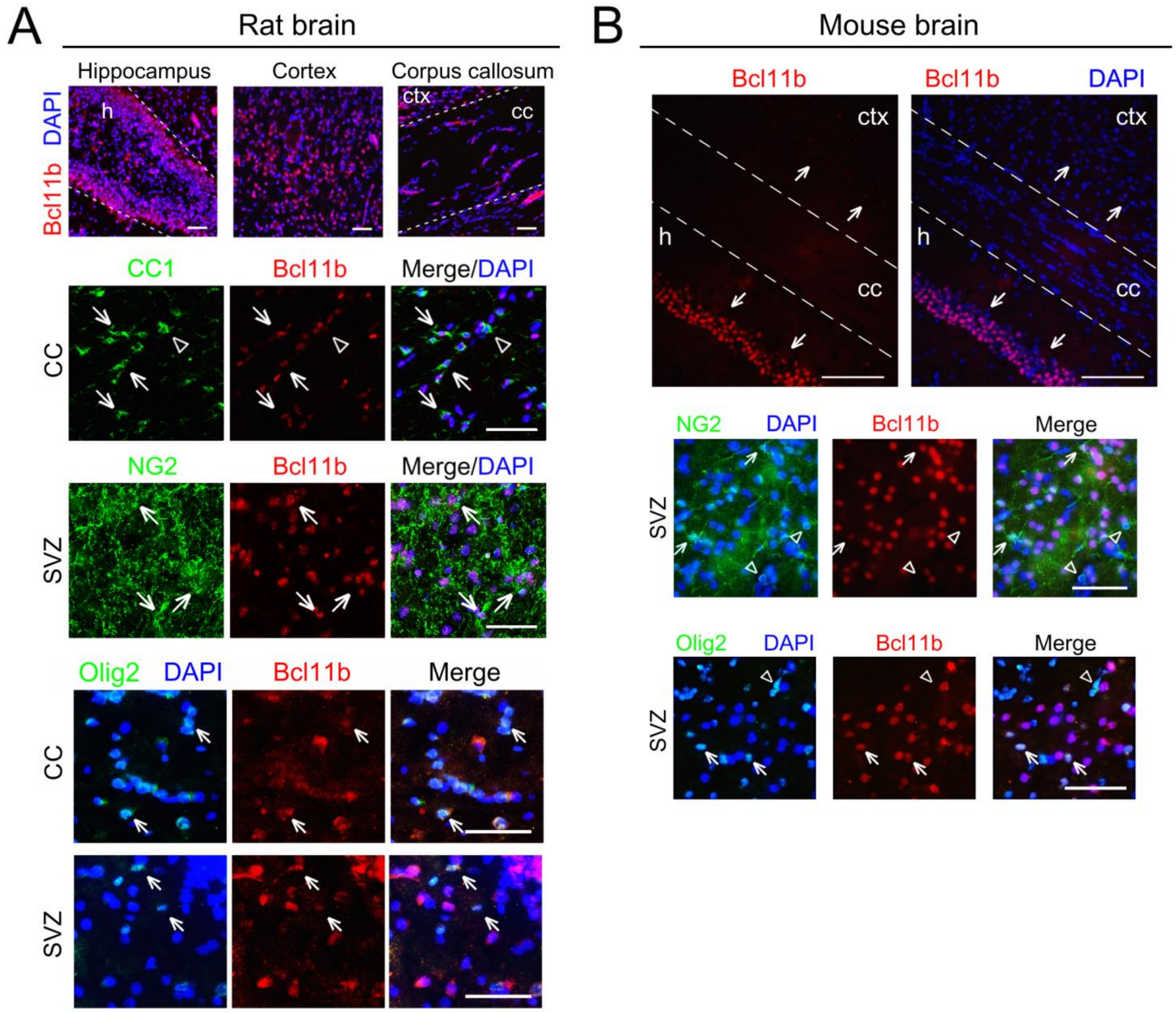


**Supplementary Figure 1. Bcl11b expression in the rat and mouse brain** (A) The brain tissues prepared from adult male rats were sectioned and subjected to immunofluorescence for Bcl11b. In the upper panel, Bcl11b-positive immunreactivity (red) was detected in the nucleus of the neural cells in the dentate gyrus of hippocampus (h), cortex (ctx), and corpus callosum (CC). In the middle and lower panels, double immunofluorescence indicated that Bcl11b expression (red) was detectable in $CC1^+$ oligodendrocytes, $NG2^+$ glial progenitors, and $Olig2^+$ oligodendroglial lineage cells (green) in CC and subventricular zone (SVZ; arrows), respectively. (B) The brain tissues prepared from adult male mice were sectioned, and subjected to immunofluorescence for Bcl11b. In the upper panel, neurons in the hippocampus the cortex with Bcl11b expression were pointed by arrows. We noticed that Bcl11b expression was rarely detected in CC. In the lower panel, double immunostaining was performed to identify $NG2^+$ glial progenitor's cells and $Olig2^+$ oligodendroglial lineage cells (green) in the SVZ with or without Bcl11b expression (red). The $Bcl11b^+$/$NG2^+$- and $Bcl11b^+$/$Olig2^+$-cells were indicated by arrows, whereas $Bcl11b^-$/$NG2^+$- or $Bcl11b^-$/$Olig2^+$-cells were showed by arrowhead. Scale bar, 50 μm.
