# Supplementary material for: Function of B-Cell CLL/Lymphoma 11B in Glial Progenitor Proliferation and Oligodendrocyte Maturation
Source: Front Mol Neurosci. 2018 Jan 24;11:4. doi: 10.3389/fnmol.2018.00004 (PMC5787563; doi:10.3389/fnmol.2018.00004)
Supplement: Supplementary file 2 [file Presentation_2.PDF]

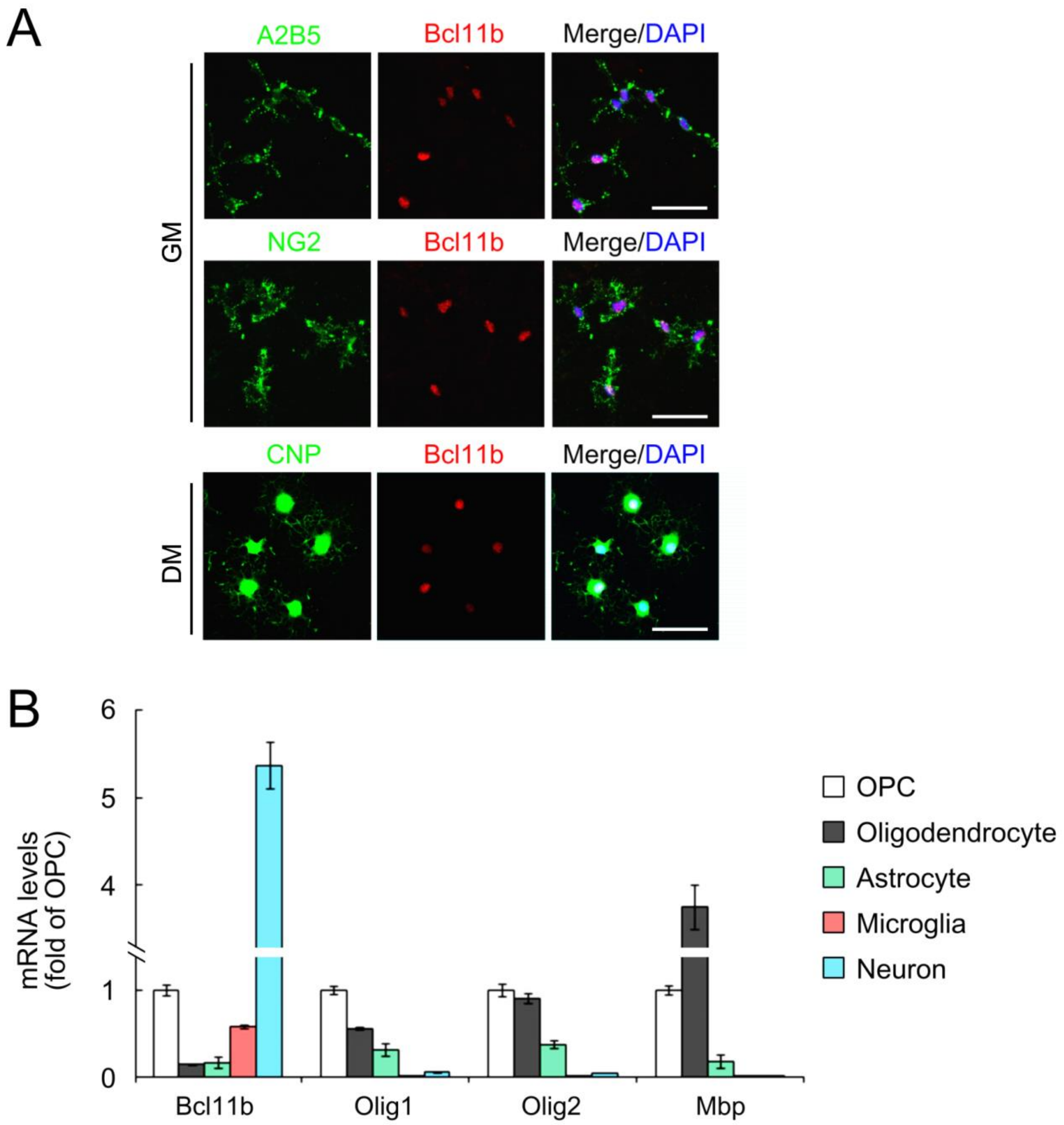

Supplementary Figure 2. Bcl11b expression in rat glial progenitors and in rat CNS neural cells. (A) Rat glial progenitor cells (GPCs) that were maintained in growth medium (GM) for 2 days and differentiation medium (DM) for 5 days were subjected to immunofluorescence for glial progenitor cell markers (A2B5 and NG2) and OLG differentiation marker (CNPase). Scale bar, 50 μm. (B) Oligodendrocyte precursor cells (OPCs) and oligodendrocytes were derived from neural stem cells prepared from rat embryonic 14.5 day. Primary neural cells (neurons, astrocytes, and microglia) were prepared from the brain tissue of P0-1 rat pup, and subjected to QPCR analysis for the measurement of Bcl11b, Olig1, Olig2, and Mbp. Data are presented as means ± SEM of at least three independent experiments.
