# Supplementary material for: Function of B-Cell CLL/Lymphoma 11B in Glial Progenitor Proliferation and Oligodendrocyte Maturation
Source: Front Mol Neurosci. 2018 Jan 24;11:4. doi: 10.3389/fnmol.2018.00004 (PMC5787563; doi:10.3389/fnmol.2018.00004)
Supplement: Supplementary file 3 [file Presentation_3.PDF]

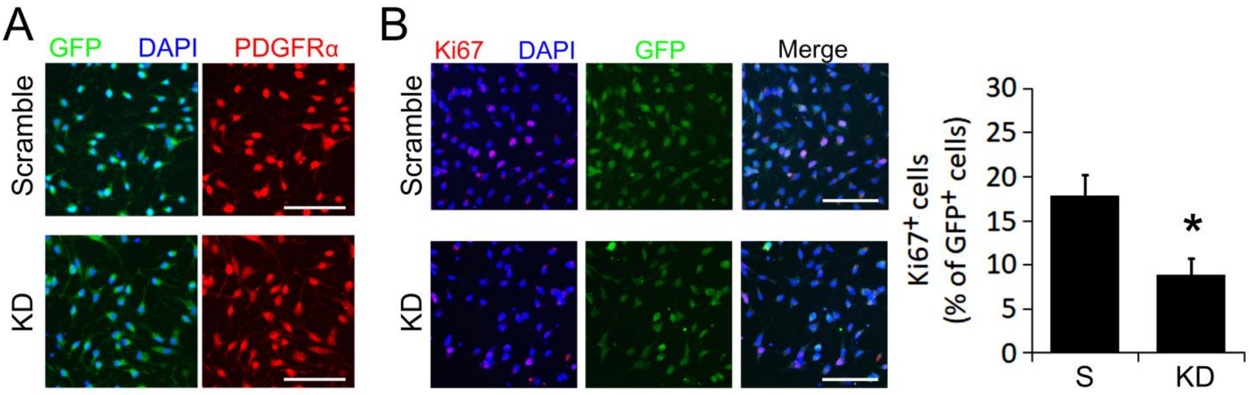

Supplementary Figure 3. Decreased proliferation level of rat OPCs with Bcl11b-KD. OPCs were derived from primary NSCs as described in Materials and Methods. OPCs were infected by lenti-ctrl (scramble; S) and lenti-sh-Bcl11b (KD) and maintained in GM with puromycin for 2 days. The cultures were then subjected to immunofluorescence for PDGFRα (A) or Ki67 (B). OPCs were identified by examining PDGFRα in both scramble and Bcl11b-KD cultures. The results indicated that ~95% PDGFRα$^+$/GFP$^+$-cells were observed in both cultures, pointing to a high purity of OPCs in the cultures we used. In addition, the proliferation of OPCs was estimated by measuring the proportion of Ki67$^+$/GFP$^+$-OPCs. Data are presented as means ± SEM of three independent experiments. *$p < 0.05$ versus the scramble. Scale bar, 50 μm.
