# Supplementary material for: Function of B-Cell CLL/Lymphoma 11B in Glial Progenitor Proliferation and Oligodendrocyte Maturation
Source: Front Mol Neurosci. 2018 Jan 24;11:4. doi: 10.3389/fnmol.2018.00004 (PMC5787563; doi:10.3389/fnmol.2018.00004)
Supplement: Supplementary file 4 [file Presentation_4.PDF]

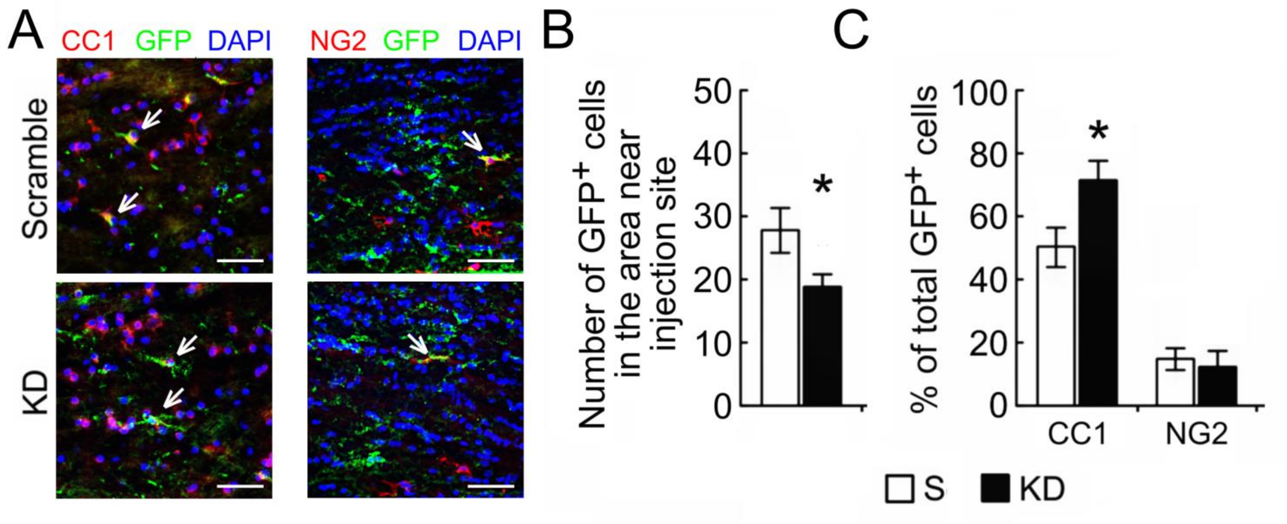

Supplementary Figure 4. Induction of mature oligodendrocytes from implanted GPCs with Bcl11b-KD. The adult rats were treated with lysolecithin for 3 days followed by the implantation of scramble GPCs (S) and Bcl11b-KD GPCs (KD) into the cortical area just above the corpus callosum. The brain sections were collected after 11dpi, and subjected to immunofluorescence for CC1 and NG2. The percentage of $GFP^+/CC1^+$- and $GFP^+/NG2^+$-cells over total GFP^+ cells were calculated. The GFP^+-cells that migrated from the injection site to the adjacent area (0.04 mm^2/observation field) were counted. Data are presented as means ± SEM from at least 3 animals in each group. *$p < 0.05$ versus scramble. Scale bar, 50 μm.
